# Ischemic preconditioning of the muscle reduces the metaboreflex response of the knee extensors

**DOI:** 10.1007/s00421-021-04815-0

**Published:** 2021-10-01

**Authors:** Luca Angius, Benjamin Pageaux, Antonio Crisafulli, James Hopker, Samuele Maria Marcora

**Affiliations:** 1 Faculty of Health and Life Sciences, Department of Sport, Exercise and Rehabilitation, Northumbria University, Newcastle upon Tyne, UK; 2 Endurance Research Group, School of Sport and Exercise Sciences, University of Kent, Chatham Maritime, UK; 3 École de Kinésiologie et des Sciences de l’Activité Physique (EKSAP), Faculté de Médicine, Université de Montréal, Montréal, QC Canada; 4 Centre de Recherche de l’Institut Universitaire de Gériatrie de Montréal (CRIUGM), Montréal, QC Canada; 5 The Department of Medical Sciences, Sports Physiology Laboratory, University of Cagliari, Cagliari, Italy; 6 Department of Biomedical and NeuroMotor Sciences (DiBiNeM), University of Bologna, Bologna, Italy

**Keywords:** Ischemic preconditioning, Metaboreflex, Exercise, Performance, Pain, Afferent feedback

## Abstract

**Purpose:**

This study investigated the effect of ischemic preconditioning (IP) on metaboreflex activation following dynamic leg extension exercise in a group of healthy participants.

**Method:**

Seventeen healthy participants were recruited. IP and SHAM treatments (3 × 5 min cuff occlusion at 220 mmHg or 20 mmHg, respectively) were administered in a randomized order to the upper part of exercising leg’s thigh only. Muscle pain intensity (MP) and pain pressure threshold (PPT) were monitored while administrating IP and SHAM treatments. After 3 min of leg extension exercise at 70% of the maximal workload, a post-exercise muscle ischemia (PEMI) was performed to monitor the discharge group III/IV muscle afferents via metaboreflex activation. Hemodynamics were continuously recorded. MP was monitored during exercise and PEMI.

**Results:**

IP significantly reduced mean arterial pressure compared to SHAM during metaboreflex activation (mean ± SD, 109.52 ± 7.25 vs. 102.36 ± 7.89 mmHg) which was probably the consequence of a reduced end diastolic volume (mean ± SD, 113.09 ± 14.25 vs. 102.42 ± 9.38 ml). MP was significantly higher during the IP compared to SHAM treatment, while no significant differences in PPT were found. MP did not change during exercise, but it was significantly lower during the PEMI following IP (5.10 ± 1.29 vs. 4.00 ± 1.54).

**Conclusion:**

Our study demonstrated that IP reduces hemodynamic response during metaboreflex activation, while no effect on MP and PPT were found. The reduction in hemodynamic response was likely the consequence of a blunted venous return.

## Introduction

Ischemic preconditioning (IP) is a non-invasive procedure involving three to four bouts of non-lethal ischemia commonly administered via 5 min cycles of circulatory occlusion and reperfusion by inflating and gradually deflating a blood pressure cuff placed around the limb. IP has been shown to confer protection to myocardial tissue and skeletal muscle to subsequent ischemic and reperfusion injury (Tapuria et al. 2008). Experimental studies described IP as a safe method to reduce infarct size and cardiac dysfunction in patients with stable angina during exercise (Kharbanda et al. 2002; Crisafulli et al. 2004b). From a physiological perspective, IP stimuli result in complex intracellular signals, many of which converge on the mitochondria (Yellon and Downey 2003; Hausenloy and Yellon 2008; Heusch et al. 2008), but the exact underlying mechanisms are still unclear and are currently under investigation. Experimental studies reported beneficial effects on cardiac and skeletal muscle following IP administration. Specifically, IP reduces cardiac tachyarrhythmias (Oxman et al. 1997), myocardial infarct size (Kharbanda et al. 2002) and ischemia–reperfusion injury in clinical settings (Loukogeorgakis et al. 2005). Experimental studies investigating the effect of IP in the muscle reported an increase in PCr production and higher oxygen consumption (Andreas et al. 2011), reduced glycogen depletion (Lintz et al. 2013), reduced lactate production (Addison et al. 2003) and attenuated ischemia-induced mitochondrial dysfunction (Mansour et al. 2012).

A growing number of experimental studies demonstrated a positive effect of IP on subsequent endurance performance measured during various tasks such as cycling, running, and swimming (Incognito et al. 2016; Caru et al. 2019). The mechanisms through which IP might induce an ergogenic effect are still unclear and numerous hypotheses are proposed to explain its positive effect on endurance performance. Previous studies reported increased local blood flow (Riksen et al. 2004), oxygen delivery (Saito et al. 2004) and decreased lactate accumulation (Bailey et al. 2012). Alternatively, other authors proposed that the ergogenic effect of IP might rely on the desensitization/defunctionalization of the metabo-nociceptive afferent neurons commonly known as group III/IV muscle afferents (Crisafulli et al. 2011b; Cruz et al. 2016).

Group III/IV muscle afferents are free nerve endings able to detect variations of the mechanical and metabolic status of the muscle (i.e., mechano-metabo receptors) (Rowell and O’Leary 1990 ). The neural feedback arising from group III/IV muscle afferents is known to increase cardiovascular responses. This specific response is called exercise pressor reflex or mechano-metaboreflex, and is essential for the regulation of cardiovascular response during physical exercise (Rowell and O’Leary 1990 ; Amann et al. 2011). Group III/IV muscle afferents are also involved in the neurophysiology of muscle pain as they ensure the detection of noxious stimuli (i.e., nociceptors) and transmit this information through the spinal cord to higher brain centres to generate muscle pain (O’Connor and Cook 1999). Discharge of group III/IV muscle afferents has been demonstrated to increase with level of metabolites (Kaufman 2012 ) and the exercise intensity (Crisafulli et al. 2006, 2008), and this discharge is directly alters cardiovascular responses and muscle pain (O’Connor and Cook 1999).

To the best of our knowledge, only two studies have investigated the effect of IP on discharge from group III/IV muscle afferents. Mulliri et al. (2016) reported a reduction in hemodynamic response during metaboreflex activation when IP was administered prior to dynamic handgrip exercise. Conversely, Incognito et al. (2017) reported no effect of IP on metaboreflex activation and muscle sympathetic responses to static handgrip task. Other researchers suggested that the ergogenic effect of IP might rely on the reduction of muscle pain intensity during exercise (Caru et al. 2019). Franz et al. (2018) reported a reduction in pain intensity after the 24, 48 and 72 h when IP was administered prior to eccentric exercise of the bicep brachii muscles. As these studies reported a discrepancy in their results, further investigations are necessary to clarify whether IP alters the discharge from group III/IV muscle afferents measured indirectly via the evaluation of the metaboreflex.

Although the use of protocols involving small muscle mass provided important preliminary findings on the effect of IP on feedback from group III/IV muscle afferents, the use of protocol involving large muscle mass may have the advantage of inducing greater cardiovascular responses to the exercise, thus potentially maximizing the effect of IP on cardiovascular responses to the task performed. In this regard, data from our research group demonstrated a greater hemodynamic response during exercise and metaboreflex activation on cycling and running exercise compared to intermittent handgrip exercise (Crisafulli et al. 2004a, 2008). In addition, as neurophysiological responses to physical exercise differs between various muscle groups (Gandevia 2001; Sidhu et al. 2013), therefore it is possible that IP applied on large muscle such as the knee extensors might have different effect on group III/IV muscle afferents compared to IP applied on small muscle group such as the forearm muscles. In addition, given the interest of researchers on the application of IP for the improvement in exercise performance, the application on locomotor muscles such as the knee extensors, might provide interesting insight for real world applications. Indeed, the knee extensors play an important role in locomotion and antigravity activities such as standing, walking, and running as well as requiring a higher metabolic and neuromuscular demand compared to the forearm musculature.

To the best of our knowledge, no study has investigated the effect of IP on group III/IV muscle afferents arising from the locomotor muscles during metaboreflex activation, sensitivity of group III/IV muscle afferents and muscle pain. Considering this gap in the literature, the present study aimed at applying IP to the knee extensor muscles to investigate its effect on metaboreflex activation and muscle pain during unilateral dynamic leg extension exercise in a group of healthy volunteers.

## Material and methods

### Study population

Seventeen healthy participants, 13 males (mean ± SD, 25.4 ± 4.6 years; 181.7 ± 7.1 cm height; body mass 76.7 ± 9.9 kg) and 4 females (mean ± SD, 25.7 ± 2.50 years; 169.2 ± 5.6 cm height; body mass 60.7 ± 4.7 kg) participated in this study. Based on G*Power software calculation, this sample allows us to detect a significant treatment difference in MAP of at least 5 mmHg, with a power of 0.95 and with a two-sided significance level of 0.05. All participants were physically active and free from any history of cardiorespiratory disease or was taking any medication at the time of the study. Physically active was defined as performing moderateto high-intensity exercise at least twice a week for a minimum of 6 months. Based on the amount and intensity of physical activity performed per week, our participants can be included in the performance level 2 according to the classification given by de Pauw et al. (2013). All the female participants were using monophasic oral contraceptive pills. To reduce the effect of endogenous hormones on the cardiovascular response, the testing sessions were performed in the 21-day consumption period of the pill (Ansdell et al. 2019). Written consent was obtained from all participants following explanation of the study during the familiarization visit.

### Experimental design

**Fig. 1:**
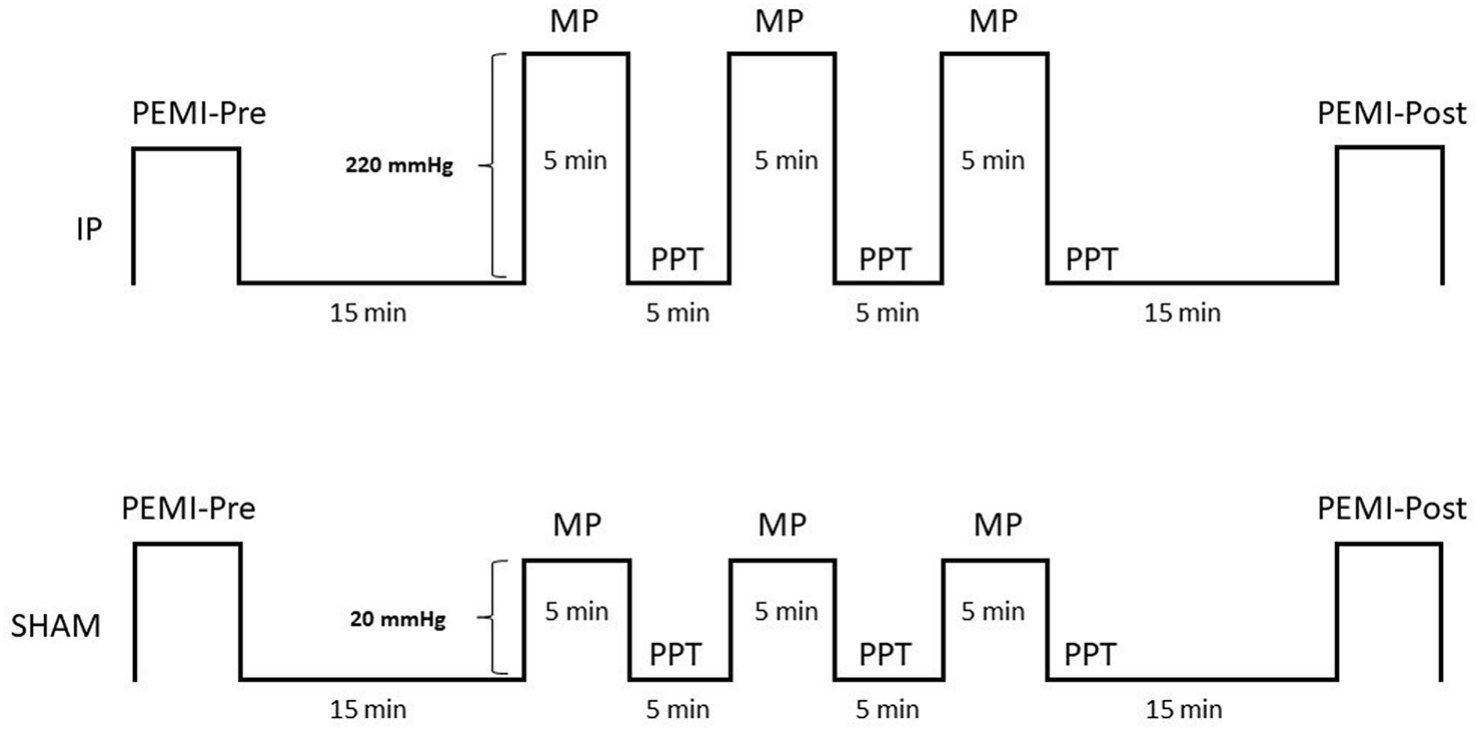
Schematic diagram of all experimental procedures, during ischemic preconditioning (IP) or sham (SHAM) treatment. Post-exercise muscle ischemia (PEMI) protocol, pain pressure threshold (PPT) test, and muscle pain (MP) during IP or SHAM treatment

Participants visited the laboratory on three different occasions using a within-subjects, crossover, randomized experimental design. During the first visit, volunteers were familiarized with all the experimental procedures. Participants visited the laboratory on two further occasions in a randomized order to undertake either ischemic preconditioning (IP) or SHAM treatment (see Fig. 1). Participants were given instructions to avoid caffeine, alcohol, autonomic nervous system stimulants or depressants, and strenuous exercise for 72 h prior to each visit. All experimental sessions were carried out in a temperature-controlled, air-conditioned room (22 °C; relative humidity between 40–50%) at the same time of the day with at least 72 h recovery between-sessions.

### Familiarization

During this first visit, each participant filled a medical questionnaire (Thomas et al. 1992), and signed an informed consent form. A one leg-dynamic knee extension test of the participant’s dominant leg was performed to assess their maximum achievable workload achievable (*W*_max_). Participants were seated on a leg-extension machine (Body-Solid GCEC340, Body-Solid Inc.) with the upper part of the leg horizontal and the knee flexed at 90°. The machine arm was attached approximately 1 cm above the malleoli of the exercising leg and the non-exercising leg was maintained at 90° knee flexion. During the test participants were asked to extend their exercising leg from 90° knee flexion to 150° knee extension at a rate of 30 repetitions/min indicated by a digital metronome. Each cycle involved 1 s for the concentric and the eccentric phase. The test started with a workload at 2.5 kg and increased by 2.5 kg/min until volitional exhaustion. A 2.5 kg weight plate was manually added by the researchers in the machine arm at the end of each minute. Volitional exhaustion was defined as the point at which the participant was no longer able to maintain a cadence of 30 repetitions/min for more than 5 s despite strong verbal encouragement.

### Ischemic preconditioning and sham treatments

A blood pressure cuff was placed around the upper part of the exercising leg’s thigh and rapidly inflated by an automated pneumatic device (Hokanson E20 Rapid Cuff Inflator and AG101 Air Source, Bellevue, WA). In the IP condition, the cuff was inflated up to 220 mmHg, while in the SHAM condition the pressure was kept at 20 mmHg to not alter leg circulation (Crisafulli et al. 2011b; Bailey et al. 2012). In both visits, the cuff was kept inflated and deflated for 5 min periods and repeated three times with the participant in a supine position (Crisafulli et al. 2011b; Bailey et al. 2012).

### Post-exercise muscle ischemia (PEMI) protocol

This protocol involved 3 min of resting, followed by 3 min of exercise consisting of rhythmic dynamic contraction of the exercising leg at 70% of *W*_max_ (30 repetitions/min controlled by a digital metronome). The exercise phase was followed by 3 min of occlusion of the exercising leg. Occlusion was induced immediately after exercise termination by a rapid (< 5 s) inflation of a cuff placed in the upper part the exercising leg’s thigh to 50 mmHg above peak systolic pressure recorded during exercise. PEMI protocol was repeated before (PEMI-Pre) and 15 min after IP or SHAM treatment (PEMI-Post). This protocol has been widely demonstrated to elicit substantial hemodynamic response during metaboreflex activation (Rowell and O’Leary 1990; Boushel 2010) as well as to monitor the activity of muscle afferents in healthy and symptomatic populations (Rowell and O’Leary 1990; Boushel 2010; Crisafulli 2017). Additionally, PEMI protocol allows researchers to isolate the metaboreflex mediating cardiovascular responses from central command and mechanoreflex activation, since these two cardiovascular mechanisms no longer operate in this setting (Rowell and O’Leary 1990; Boushel 2010). To date, there is no possibility to assess non-invasively activity from group III/IV muscle afferents in humans. Therefore, we used variations in mean arterial pressure (MAP) as marker of feedback from group III/IV muscle afferents as performed in a previous study (Pageaux et al. 2015).

### Hemodynamic variables

Hemodynamic variables were recorded throughout all phases of the PEMI protocols. A transthoracic bioimpedance device (Physioflow PF05L1, Manatec, Petit-Ebersviller, France) allowing continuous non-invasive monitoring of heart rate (HR), stroke volume (SV), cardiac output (CO), left ventricular ejection time (LVET), stroke volume and left ventricular ejection time ratio (SV/LVET), end diastolic volume (EDV) and systemic vascular resistance (SVR) was used. The SV/LVET ratio was considered an index of contractility performance (Tanaka et al. 1986; Gledhill et al. 1994). The impedance method assumes that when an electrical current circulates through the chest, the aortic blood flow induces variations in electrical conductivity. Thus, changes in the transthoracic electrical impedance during cardiac phases are representative of changes in SV; the method has been previously described by Charloux et al. (2000). Three sets of two electrodes (Ambu Blue Sensor VL, Ambu A/S, Ballerup, Denmark) were placed on the supraclavicular fossa at the left base of the neck, in correspondence to the V1 and V6 positions close to the left ventricle to obtain an ECG signal, and then on the back in the midpoint of the spine corresponding to the same vertical position as the xiphoid process. Skin placement areas were shaved and cleaned. Before each test, the Physioflow was calibrated using a standardized procedure based on 30 consecutive heartbeats while the participant was in a seated position. Arterial blood pressure was monitored by an automated blood pressure device (Tango^+^, SunTech Medical, Morrisville, NC) (Cameron et al. 2004; Hartwich et al. 2011) with a set of three electrodes placed in the V2, V6 and RL positions. The cuff was placed on around the left arm of the subject. At the end of each minute during the post-exercise muscle ischemia session, systolic arterial pressure (SAP), diastolic arterial pressure (DAP) and MAP were recorded. MAP was calculated as follows:

$${\text{MAP}} = \frac{{\left( {2 \times {\text{DAP}}} \right) + {\text{SAP}}}}{3} .$$

### Pain measurement

Muscle pain intensity during the IP and SHAM treatment, during exercise and PEMI manoeuvre was quantified with the scale developed by Cook et al. (1997). Pain is commonly used in the psychophysiological literature as an indirect marker of group III/IV afferents (O’Connor and Cook 1999; de Morree et al. 2012; Pageaux et al. 2015). Participants were required to verbally report their muscle pain intensity according to a 0–10  point scale. Standardized instructions were provided to the participants at the beginning of each visit to ensure the proper use of the scale. Briefly, participants were asked to report “the intensity of hurt they feel in their quadriceps only” (Cook et al. 1997). Muscle pain intensity was rated at the end of each min during PEMI protocols and all the three cycles of occlusion while receiving IP or SHAM treatment.

Pain threshold reflects the detection of the minimum intensity of a stimulus generating a painful sensation (IASP). Any change in pain threshold reflects, albeit indirectly and with some limitations, the change in sensitivity of group III/IV muscle afferents. Therefore, any change in pain pressure threshold can be used an indirect marker of change in sensitivity of group III/IV muscle afferents (Graven-Nielsen et al. 2004; Schabrun et al. 2016; De Martino et al. 2018). Sensitivity of group III/IV muscle afferents was quantified by means of pain pressure threshold (PPT was assessed alternatively in both thighs (exercising vs non-exercising leg) after each cycle of occlusion during IP and SHAM treatment by a pressure algometer (Force Ten FDX 50, Wagner Instruments, Greenwich, CT). The force was gradually increased (5 N·s^−1^) in three different points located in the middle part of the rectus femoris, where the blood pressure cuff was placed for the IP and SHAM treatment. Each point was marked to apply the algometer in the same location. The rubber footplate of the algometer was held perpendicular to the muscle and the display was turned away from the participants. Participants were instructed to report a change from the sensation of pressure to the appearance of a slightly uncomfortable pain. The average of the two nearest of three measured values was considered as the PPT (de Morree et al. 2012).

### Perception of effort measurement

Perception of effort was measured at the end of each minute during the exercise phase of PEMI protocols by using the 6–20 Borg scale (Borg 1998). Standardized instructions were provided to participants at the beginning of each visit to ensure proper use of the scale. Briefly, subjects were asked to rate how hard they were driving their leg during the exercise (Marcora 2010). Subjects were also asked to not use this rating as an expression of leg muscle pain (i.e., the intensity of hurt that a participant feels in his quadriceps muscles only).

### Statistical analysis

All data are presented as mean ± SD. Assumption for statistical analysis such as normal distribution was checked by the Shapiro–Wilk test while sphericity of data was checked by using the Mauchly's test. The Greenhouse–Geisser correction to the degrees of freedom was applied when violation to sphericity was found. Data from hemodynamic variables were averaged for 3 min during each phase of the PEMI protocols and then analyzed separately for each period by means of two-way repeated measures ANOVA (factors condition: IP vs SHAM and test: PEMI-Pre vs PEMI-Post). Muscle pain intensity during PEMI protocols was averaged for 3 min and compared by means of two-way repeated measures ANOVA (factors condition: IP vs SHAM and test: PEMI-Pre vs PEMI-Post). Muscle pain intensity during IP and SHAM treatment was averaged for 5 min and then compared by means of two-way repeated measures ANOVA (factors condition: IP vs SHAM and occlusion: 1, 2 and 3). PPT was analyzed by means of three-way repeated measures ANOVA (factors condition: IP vs SHAM, leg: exercising vs non-exercising and occlusion: 1, 2 and 3). Significant main effect or interaction was followed by Bonferroni post hoc test when appropriate. Statistical analysis was carried out by using commercially available software (IBM, SPSS Statistics 23.0). Significance was set as *p* < 0.05. Partial eta squared (*η*^2^_p_) are reported, and thresholds for small, moderate, and large effects were set at 0.01, 0.07, and 0.14, respectively (Cohen 2013). For paired comparisons, Cohen’s *d*_z_ were calculated using G*Power software (version 3.1.6, Universität Düsseldorf, Germany) and thresholds for small, moderate and large effects were set at 0.2, 0.5, and 0.8 respectively (Cohen 2013).

## Results

All participants completed the study and none of them reported any complication during the ischemic preconditioning treatments and during the PEMI protocol. The *W*_max_ reached by participants during the familiarization visits was 21.62 ± 6.43 kg. No statistical differences were found between the two conditions for the hemodynamic variables measured during the two resting phases (all *p* > 0.12 and all *η*^2^_p_ < 0.142). Therefore, participants performed the PEMI protocols starting with similar hemodynamic levels. In addition, this data also indicates that IP did not affect hemodynamic variables at rest (see Table 1).

**Table 1:** Hemodynamic variables during the PEMI protocols at rest and during exercise phases in both conditions

	SHAM		IP	
	PEMI-Pre	PEMI-Post	PEMI-Pre	PEMI-Post
HR (bpm)				
Rest	68.49 ± 10.69	66.69 ± 10.73	68.57 ± 10.01	67.87 ± 7.23
Exe	98.54 ± 11.41^#^	97.77 ± 11.30^#^	101.30 ± 12.33^#^	101.75 ± 8.79^#^
SV (ml)				
Rest	75.37 ± 5.63	74.19 ± 4.92	74.07 ± 4.59	74.39 ± 3.47
Exe	87.57 ± 9.49^#^	91.24 ± 14.64^#^	93.37 ± 15.18^#^	89.07 ± 9.28^#^
CO (l·min^−1^)				
Rest	5.17 ± 0.96	4.93 ± 0.73	5.09 ± 0.87	5.05 ± 0.57
Exe	9.08 ± 1.37^#^	9.54 ± 1.77^#^	9.15 ± 1.487^#^	9.09 ± 1.09^#^
EDV (ml)				
Rest	110.40 ± 15.40	111.35 ± 15.73	110.16 ± 10.16	109.09 ± 10.20
Exe	113.17 ± 15.92	112.89 ± 16.14	111.05 ± 10.49	103.07 ± 25.29
LVET (ms)				
Rest	281.36 ± 86.44	276.74 ± 76.74	287.71 ± 76.21	293.39 ± 85.46
Exe	240.43 ± 69.50^#^	240.29 ± 70.11^#^	238.28 ± 75.61^#^	246.94 ± 78.98^#^
SV/LVET				
Rest	0.41 ± 0.08	0.39 ± 0.07	0.37 ± 0.08	0.40 ± 0.08
Exe	0.30 ± 0.06^#^	0.32 ± 0.06^#^	0.29 ± 0.05^#^	0.28 ± 0.05^#^
SVR (dyne·s^−1^·cm^−5^)				
Rest	1346.54 ± 230.05	1417.09 ± 173.36	1350.68 ± 180.73	1369.77 ± 172.41
Exe	882.54 ± 173.64^#^	853.85 ± 219.81^#^	922.60 ± 210.78^#^	920.10 ± 160.12^#^
SAP (mmHg)				
Rest	116.82 ± 10.06	118.82 ± 10.21	119.27 ± 11.61	119.73 ± 10.98
Exe	148.04 ± 15.80^#^	146.95 ± 10.88^#^	152.61 ± 15.04^#^	149.61 ± 13.52^#^
DAP (mmHg)				
Rest	67.69 ± 9.23	68.55 ± 8.32	66.12 ± 11.06	69.41 ± 11.31
Exe	78.50 ± 8.92^#^	81.07 ± 10.66^#^	79.65 ± 9.84^#^	77.90 ± 9.08^#^
MAP (mmHg)				
Rest	84.07 ± 7.00	85.31 ± 6.04	83.84 ± 7.44	86.18 ± 9.24
Exe	101.68 ± 7.64^#^	103.03 ± 9.03^#^	103.97 ± 8.54^#^	101.80 ± 7.89^#^

Values are means ± SD of rest and PEMI condition during both conditions of heart rate (HR), stroke volume (SV), cardiac output (CO), end diastolic volume (EDV), left ventricular ejection time (LVET), stroke volume/left ventricular ejection time ratio (SV/LVET), systemic vascular resistance (SVR), systolic arterial pressure, (SAP), diastolic arterial pressure (DAP) and mean arterial pressure (MAP)

^#^Significantly different compared to Rest (*p* < 0.05)

### Hemodynamic response during exercise

No condition × time interaction was found for all the hemodynamic variables (all *p* > 0.104 and all *η*^2^_p_ < 0.157). HR and SV, SV/LVET significantly increased compared to resting phase (all *p* < 0.001 and all *η*^2^_p_ > 0.908), which led to a significant increase in CO compared to resting phase (all *p* < 0.001 and all *η*^2^_p_ > 0.908). There was no difference between conditions (all *p* > 0.413 and all *η*^2^_p_ < 0.042). LVET, and SVR significantly decreased compared to resting phase (all *p* < 0.001 and all *η*^2^_p_ > 0.795), while no difference was found for EDV (all *p* > 0.423 and all *η*^2^_p_ < 0.041). Blood pressure response was significantly affected by the exercise phase and resulted in an increase of SAP, DAP and MAP (all *p* < 0.001 and all *η*^2^_p_ > 0.938).

### Hemodynamic response during the occlusion phase

**Fig. 2:**
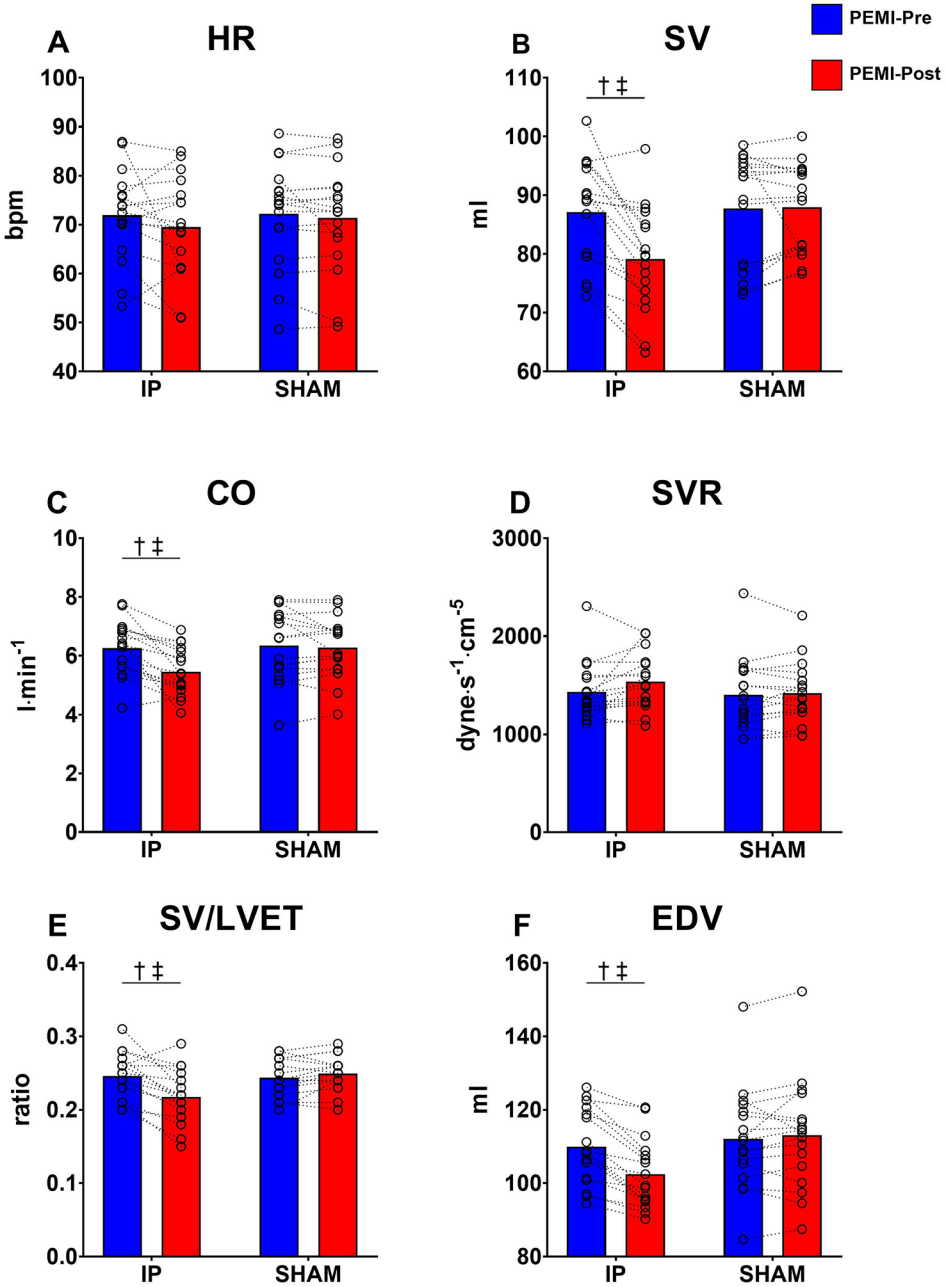
Hemodynamic values gathered during the occlusion phase of PEMI protocols. Panel **A** shows heart rate (HR). Panel **B** shows stroke volume (SV). Panel **C** shows cardiac output (CO). Panel **D** shows systemic vascular resistance (SVR). Panel **E** stroke volume and left ventricular ejection time ratio (SV/LVET). Panel **F** shows end diastolic volume (EDV). †Denotes significant condition × time interaction (*p* < 0.05); ‡Denotes significant difference at PEMI-Post in IP (*p* < 0.05). Values are presented as mean ± SD (*n* = 17)

**Fig. 3:**
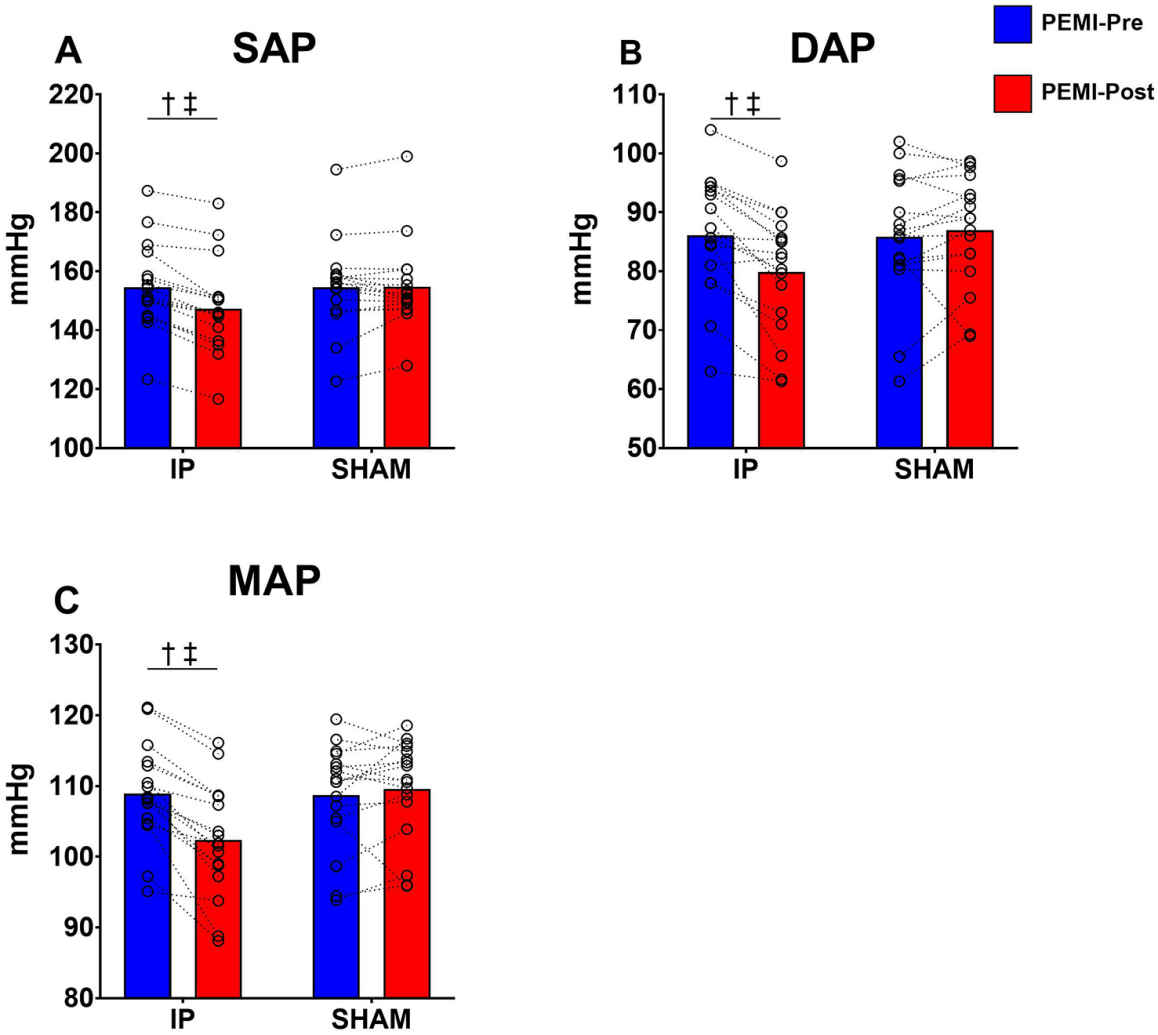
Hemodynamic values gathered during the occlusion phase of PEMI protocols. Panel **A** systolic arterial pressure (SAP). Panel **B** shows diastolic arterial pressure (DAP). Panel **C** shows mean arterial pressure (MAP). †Denotes significant condition × time interaction (*p* < 0.05); ‡Denotes significant difference at PEMI-Post in IP condition (*p* < 0.05). Values are presented as mean ± SD (*n* = 17)

A significant condition × time interaction was found for SV, CO, SV/LVET, EDV, SAP, DAP and MAP (all *p* < 0.05 and all *η*^2^_p_ > 0.402). Follow-up tests revealed a significant decrease of SV (*p* = 0.037, *d*_z_ = 1.246), CO (*p* = 0.045, *d*_z_ = 1.143), SV/LVET (*p* = 0.043, *d*_z_ = 1.278), EDV (*p* = 0.021, *d*_z_ = 1.652), SAP (*p* < 0.001, *d*_z_ = 2.111), DAP (*p* = 0.03, *d*_z_ = 1.281) and MAP (*p* = 0.015, *d*_z_ = 1.812) at PEMI-Post compared to PEMI-pre in the IP condition only. No significant differences were found for HR, LVET and SVR (all *p* > 0.29 and all *η*^2^_p_ > 0.07). All parameters are illustrated in Fig. 2 and 3.

### Perceptual parameters during ischemic preconditioning and sham treatments

**Fig. 4:**
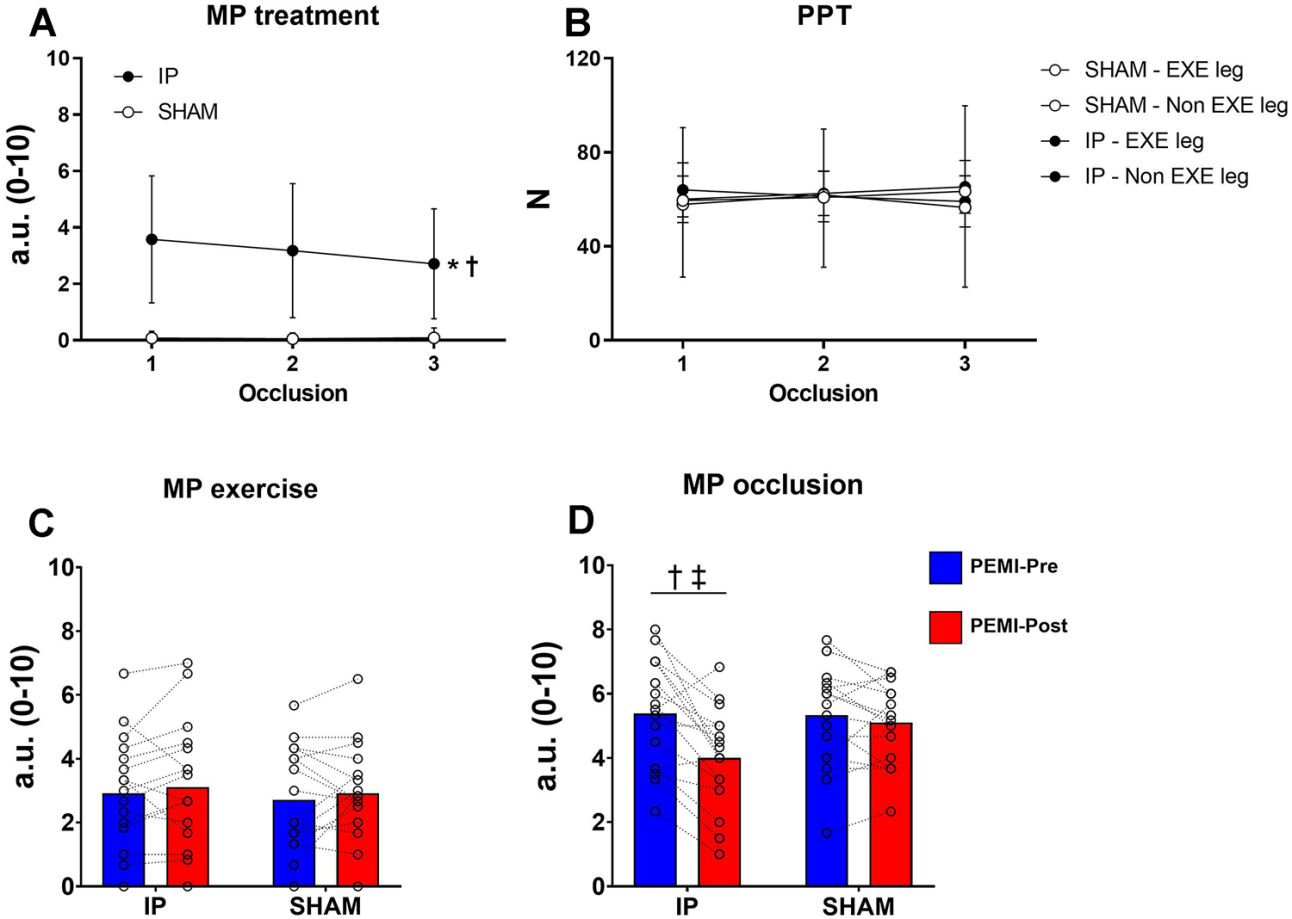
Absolute values of muscle pain intensity (MP) and pain pressure threshold (PPT) test. Panel **A** shows MP during ischemic preconditioning (IP) and sham (SHAM) treatment. Panel **B** shows PPT during ischemic preconditioning (IP) and sham (SHAM) treatment. Panel **C** shows MP during the exercise phase. Panel **D** shows MP during the occlusion phase. †Denotes significant condition × time interaction (*p* < 0.05); ‡Denotes significant difference at PEMI-Post in IP (*p* < 0.05). Values are presented as mean ± SD (*n* = 17)

A condition × time interaction was found for MP (*p* = 0.016, *η*^2^_p_ = 0.271). Follow-up tests revealed that MP was significantly higher in the experimental condition compared to SHAM condition during all occlusions (all *p* < 0.001, *d*_z_ > 1.499). Statistical analysis did not reveal any condition × leg (*p* = 0.798, *η*^2^_p_ = 0.004), or condition × time (*p* = 0.917, *η*^2^_p_ = 0.005) interaction, but revealed a time × leg interaction (*p* = 0.034, *η*^2^_p_ = 0.190) for PPT. Follow-up tests failed to reveal a significant difference time effect (all *p* > 0.109). All parameters are illustrated in Fig. 4.

### Perceptual parameters during post exercise muscle ischemia protocols

No significant condition × time interaction was found for MP during the exercise phase (*p* = 0.822, *η*^2^_p_ = 0.003). Statistical analysis revealed a significant condition × time interaction for MP during the occlusion phase (*p* = 0.002, *η*^2^_p_ = 0.461). Follow-up tests revealed a significant decrease in MP during PEMI-Post in the IP condition only (*p* = 0.044, *d*_z_ = 0. 1.081). No significant condition × time interaction was found for perception of effort during exercise (*p* = 0.961, *η*^2^_p_ < 0.001). All parameters are illustrated in Fig. 4.

## Discussion

This study sought to evaluate the effect of ischemic preconditioning applied to the knee extensor muscles on metaboreflex activation and muscle pain. This study demonstrated that IP reduced the hemodynamic response and muscle pain intensity during the metaboreflex activation, but did not affect pain pressure threshold, the hemodynamic response, or muscle pain intensity during exercise.

### Hemodynamic response during exercise and metaboreflex activation

Our data show that exercise performed at 70% of *W*_max_ elicited significant changes in both central and peripheral hemodynamic compared to the resting condition. Our data shows a substantial elevation of CO, achieved by parallel increases of both SV and HR. The SV increment during exercise was the consequence of the enhancement in contractility as demonstrated by the significant decrease in SV/LVET ratio. Further, our data demonstrated that during exercise the increase in blood pressure response was mainly achieved by the rise in CO (i.e., flow increment mechanism). The hemodynamic response elicited by the exercise protocol is in agreement with previous studies involving unilateral rhythmic exercise of the knee extensor muscles (Crisafulli et al. 2006; Amann et al. 2011). In addition, our data provide further evidence on the different hemodynamic response during exercise between small and large muscle mass. Similarly to cycling and running exercise (Crisafulli et al. 2004a, 2008) the data reported in our study show that the hemodynamic response of the knee extensors is markedly higher compared to intermittent handgrip exercise (Crisafulli et al. 2011a; Roberto et al. 2012; Marongiu et al. 2013). This greater hemodynamic response further justifies the replication of previous studies investigating the effect of IP on the forearm muscles (Mulliri et al. 2016; Incognito et al. 2017) with future studies using larger muscle mass such as the knee extensors.

During metaboreflex activation, we found that the exercise performed at 70% of *W*_max_ significantly affected central but not peripheral hemodynamic response. The data of the present study are in agreement with previous findings in human and animal settings, showing that muscle metaboreflex activation enhances blood pressure response and cardiac performance (O’Leary 1993; Piepoli et al. 1996; Spranger et al. 2013). Furthermore, our results further extend previous knowledge that the metaboreflex elicited by large muscle mass can modulate the central hemodynamics (Crisafulli et al. 2006, 2008; Amann et al. 2011). More specifically, our data show a significant increase in SV during metaboreflex activation, which most likely was the consequence of an increased cardiac performance as demonstrated by the SV/LVET ratio. This response is in agreement with previous studies involving unilateral leg extension exercise (Crisafulli et al. 2006). Contrarily to SV response, HR returned toward resting condition during the occlusion phase. This different HR response is in line with previous studies involving exercise with large muscle mass (Crisafulli et al. 2006, 2008; Watanabe et al. 2010). Fisher et al. (2010, 2013) demonstrated that exercise involving large muscle mass such as cycling, but not small muscle mass, are able to maintain HR elevation during metaboreflex activation probably due to a blunted baroreflex response. Therefore, it appears that HR response during metaboreflex activation is task dependent and reflects the complex balance between the sympathetic and parasympathetic systems. Despite the decrease in HR, CO was maintained elevated during the occlusion phase which was mostly due to the significant increase in SV.

SVR decreased during exercise and the returned towards resting conditions during the occlusion phase. This behaviour suggests that the SVR in our experiment appeared to have little effect on the increase in blood pressure response. Wyss et al. (1983) suggested that the rise in blood pressure response to the metaboreflex following low or moderate exercise intensity is mainly achieved by an increase in CO, whereas peripheral vasoconstriction becomes more important at higher intensities. Indeed, our findings suggest that the increase in blood pressure response during metaboreflex activation following high-intensity exercise was achieved by a flow-mediated response via an increase of CO rather than by peripheral vasoconstriction. Further studies involving exercise with large muscle mass should be performed to clarify the mechanism involved in metaboreflex response.

### Effects of muscle ischemic preconditioning on hemodynamic response during exercise and metaboreflex activation

Previous experimental findings demonstrated that substances released by IP interact with opioid receptors in the skeletal muscle in the early phase of cardio protection (Schultz et al. 1995; Addison et al. 2003). The excitation of opioid receptors has been shown to reduce the discharge from group III/IV muscle afferents by therefore attenuating the exercise pressor reflex (Leal et al. 2013; Estrada and Kaufman 2018; Harms et al. 2018). In accordance with these findings, a reduction in the hemodynamic response following IP was expected. However, our data showed no significant changes in the hemodynamic response during exercise following IP treatment. Similar results have also been demonstrated during rhythmic handgrip exercise (Mulliri et al. 2016).

Previous studies investigating the effect of IP on oxygen update kinetics have reported contrasting findings. Specifically, Cruz et al. (2015) reported a higher V̇O_2_ slow component and V̇O_2_ peak during the constant workload cycling exercise performed at peak power output. Conversely, Kido et al. (2015) did not report significant differences in pulmonary V̇O_2_ dynamics after IP. In contrast, muscle deoxygenation dynamics from low to moderate intensity was significantly faster following IP. Kilding et al. (2018) reported a reduction in V̇O_2_ slow component during heavy intensity cycling exercise. Wiggins et al. (2019) reported a significant improvement in deoxyhemoglobin and deoxymyoglobin primary component amplitude during cycling 15% below gas exchange ratio in hypoxia following IP. Based on these studies, it is plausible that IP might also affect the kinetics of the hemodynamic response; however, to the best of our knowledge, no studies have done so. In this regard, our data do not support the notion that IP is able to affect the kinetics of the hemodynamic response. However, it should be considered that IP might affect only muscle deoxygenation kinetics without affecting hemodynamic kinetics. Indeed, a distinct response between muscle, hemodynamic and pulmonary kinetics has previously been observed at different exercise intensities, using different muscles, and following different types of experimental manipulations (Poole and Jones 2012; Grassi and Quaresima 2016). It is also important to note that the exercise protocol used in our study was not specifically designed to investigate the effect of IP on hemodynamic kinetics, and therefore it is difficult to draw conclusions on this specific mechanism.

The importance of group III/IV for an adequate cardiorespiratory response during exercise has been widely demonstrated (Rowell and O’Leary 1990). A previous study performed by Amann et al. (2011) showed a significant reduction of the hemodynamic response during leg extension exercise when the discharge from group III/IV muscle afferents was reduced by lumbar intrathecal fentanyl. Similar findings were also reported during dynamic and static knee extension exercise following curare administration (Gallagher et al. 2001). To the best of our knowledge, no studies compared that effect of fentanyl, curare, and IP on opioid receptors. However, it is likely that the effect of IP on opioid receptors by IP is weaker than fentanyl and curare. This could explain the lack of effect of IP on the hemodynamic response during exercise in our study. Taken together, these findings provided in these studies let us suggest that the exercise induced hemodynamic response is not affected by the IP.

Our data showed a significant reduction of blood pressure response during the PEMI-Post in the IP condition, while no significant changes were found in the SHAM condition. Moreover, the data suggest that the reduction in MAP was caused by a significant reduction in CO. Since no changes in HR were found between conditions, the reduction in SV following IP led to a diminished CO. Based on our data, the lower SV was most probably affected by a reduced venous return, as testified by the lower EDV. Our findings are supported by previous experimental studies involving human and animal models which demonstrated that diastolic function is essential for an adequate hemodynamic response during the metaboreflex activation (Bastos et al. 2000; Crisafulli et al. 2009; Marongiu et al. 2013). Similar studies also demonstrated that the centralization of blood volume by peripheral vasoconstriction supports ventricular performance via the Frank–Starling mechanism (Bastos et al. 2000; Crisafulli et al. 2009). As concerns ventricular performance, we observed a reduced SV/LVET following IP. Our results are in line with the concept that the reduced diastolic function was the primary cause of the reduced blood pressure response following IP. This therefore resulted in a reduced capacity to maintain SV and CO during the metaboreflex activation.

Our results are in agreement with previous studies involving dynamic handgrip exercise (Mulliri et al. 2016), but are also in contrast with previous studies involving static handgrip (Incognito et al. 2017). Specifically, during dynamic handgrip exercise, Mulliri et al. (2016) concluded that IP had the capacity to induce venous dilation, thereby blunting cardiac preload thus reducing EDV and SV. Incognito et al. (2017) suggested that the lack of effect of IP in their study was likely influenced by the fact that participants were lying in the supine position during the experiments.

The distinct effect of IP on the hemodynamic profile between the exercise and metaboreflex activation phase can be possibly explained by neural mechanisms involved between each setting. It should be considered that during exercise, along with the metaboreflex, the central command and the mechanoreflex also operate to modulate cardiovascular responses to exercise. So, it is possible that during exercise, the reduced effect on metaboreflex induced by IP was masked by the central motor command and mechanoreflex. In addition, some redundancy exists between these neural mechanisms (Nishiyasu et al. 1994) which make the specific contribution of each difficult to establish.

### Effects of muscle ischemic preconditioning on pain

Previous authors suggested that the ergogenic effect of IP might rely on the desensitization/defunctionalization of the metabo-nociceptive afferent neurons commonly known as group III/IV muscle afferents (Crisafulli et al. 2011b; Cruz et al. 2016). We did not observe any effect of IP on muscle pain intensity during exercise. In addition, by using the PPT as a marker of the sensitivity of group III/IV muscle afferents (Graven-Nielsen et al. 2004; de Morree et al. 2012), we did not observe any effect of IP on the sensitivity of group III/IV muscle afferents. By contrast, we found a significant reduction in muscle pain intensity during the occlusion phase at PEMI-Post in IP only. Muscle pain during the occlusion phase in all PEMI protocols was significantly higher than muscle pain during exercise. Previous authors demonstrated that central command may interact with afferent feedback from the exercising muscle to modulate and attenuate pain (Ray and Carter 2007). Therefore, the higher muscle pain intensity reported during the occlusion phase in all PEMI maneuvers was possibly due to the absence of central command. It is also possible that exercise might, per se, ‘distract’ participants from muscle pain, and so during the occlusion participants focused solely on the nociceptive stimuli (Linton and Shaw 2011). If this was the case, then participants would likely report higher pain values compared to those during exercise. It must also be considered that the higher muscle pain intensity during the occlusion was caused by the cuff pressure which is able to generate pain independently of the presence of the muscle metabolites and stimulation of group III/IV muscle afferents. The application of repetitive occlusions during the IP treatment might have habituated participants with the pain sensation and thus reduced muscle pain following IP (Linton and Shaw 2011).

To the best of our knowledge, only Franz et al. (2018) investigated the effect of IP on muscle pain intensity by reporting a significant reduction in muscle pain following eccentric exercise of biceps brachii after 24, 48 and 72 h. The reduction in muscle pain intensity was also associated with an attenuated decline in contractile ability of the muscle and improvement in maximal force production. The most widely supported mechanisms causing muscle pain following eccentric exercise are connective tissue damage, muscle damage, inflammation, and oxidative stress (Armstrong 1984; Smith 1991). However, those mechanisms differ from those involved during the exercise performed in our protocol and possibly explains the lack of effect of IP on muscle pain intensity during exercise. In summary, our data do not support that IP reduces muscle pain intensity during exercise nor the sensitivity of group III/IV muscle afferents to mechanical stimuli.

### Limitations of the study

We must acknowledge some limitations to the present study. It could be argued that the inclusion of a baseline assessment (PEMI-pre) prior the treatment (IP or SHAM) could induce a carryover effect. Such effect would be revealed as significant differences between pre and post-test in the SHAM condition. However, it was not the case in our study. Importantly, if it was, the statistical analysis could have accounted for it. In addition, the baseline assessment enables us to assess the baseline responses and, eventually, correct the post-test responses for any baseline difference if present (this was not the case in our study). Secondly, adding a pre-test can improve statistical power even in randomized cross over designs. So, including a pre-test does not invalidate the internal validity of our study. The only potential concern is related to external validity, and it is about a potential pre-test by treatment interaction. In other words, the effect of treatment reported may be present only when combined with the pre-test. However, in the context of our primarily physiological study, we consider such external validity issue of minor relevance and importance compared to the potential advantages in terms of the gains in statistical validity (increased power and better treatment effect estimates) obtained with the inclusion of a pre-test.

As non-invasively monitoring the discharge of group III/IV muscle afferents during exercise is complex, we decided to use a multidisciplinary approach using PEMI and muscle pain as index of group III/IV muscle afferent activity. During PEMI, central command does not operate, and so changes in MAP are thought to represent index of metaboreflex activity. However, changes in MAP during PEMI reflects the interplay between sympathetic activity induced by muscle metaboreflex and parasympathetic activation induced by the arterial baroreflex, which could in part mask the sympathetic tone. With regard to muscle pain, it has to be acknowledged that this perception could be influence by the presence of the central command as well by changes in the psychological states of participants (O’Connor and Cook 1999).

Another possible limitation of the present study is the use if impedance cardiography to monitor the cardiovascular response during exercise (Warburton et al. 1999). Cardiac MRI and transthoracic echocardiography are considered the gold standard for the estimation of ventricular volumes end ejection fractions (Theodore et al. 2007; Grothues et al. 2004) and thus the impedance cardiography could have potentially underestimated the hemodynamic function. There are also multiple factors that can generate errors and artefacts using impedance cardiography. For example, positioning and re-positioning of electrodes; electrode contact with the skin of the participant; large neck and chest movements during strenuous efforts when large muscle mass is involved. To reduce any potential source of error, participants were placed in a sitting position with no direct contact from the back support of the exercising chair to the electrodes placed on the back. The electrodes’ location was also marked to maintain the same placement during each visit. Moreover, to reduce artifacts in the signal, participants performed one leg extension exercise that did not generate large increases in ventilation or marked neck and chest movements. During rest and PEMI periods, the impedance traces were of good quality and reference points were clearly recognizable thus making the analysis reliable. Indeed, our hemodynamic profile was comparable with previous works involving impedance cardiography during single-joint and whole-body exercise tasks (Crisafulli et al. 2006, 2008).

Lastly, it must be acknowledged that there are some limitations in the formula used to calculate the MAP. This formula considers a constant ratio between DBP and SBP, which is approximately 1/3 during the systolic and 2/3 during the diastolic period. However, this ratio is not accurate when significant changes in HR occur during exercise (Moran et al. 1995; Rogers and Oosthuyse 2000), where a significant underestimation of MAP was correlated with a linear increase in HR. While the device used in our study is validated for the measurement of hemodynamic variables during physical exercise (Charloux et al. 2000; Siebenmann et al. 2015), this device does not allow to apply the specific adjustments aforementioned. Therefore, future studies should be using a different device to account for changes in diastolic and systolic periods during exercise for a more precise calculation of MAP.

### Conclusion

In conclusion, our experiment shows that ischemic preconditioning reduces hemodynamic response and muscle pain of the knee extensor muscles during metaboreflex activation. Our results suggest that the reduction in metaboreflex activation seems to be caused by a blunted cardiac preload rather than a reduction of discharge from group III/IV muscle afferents. The integrative methodology merging the metaboreflex and pain responses also questions that ischemic preconditioning might have a different impact on subset or population of group III/IV muscle afferents. Indeed, we observed a reduction of muscle pain intensity and mean arterial pressure during metaboreflex activation which is mostly elicited by metabolic stimulation, and no reduction in muscle pain induced by mechanical pressure (PPT). As group III muscle afferents predominantly transmit information related to mechanical stimuli, and group IV muscle afferents mainly transmit information on metabolic stimuli (McCord and Kaufman 2010), further studies are required to investigate whether ischemic preconditioning might differentially interact with subset of group III/IV muscle afferents. Such studies may have the potential to identify physiological mechanisms leading to an ergogenic effect of ischemic preconditioning.

## Abbreviations

CO: Cardiac output
DAP: Diastolic arterial pressure
EDV: End diastolic volume
HR: Heart rate
IP: Ischemic preconditioning
LVET: Left ventricular ejection time
MAP: Mean arterial pressure
MP: Muscle pain
PEMI: Post-exercise muscle ischemia
PPT: Pain pressure threshold
SAP: Systolic arterial pressure
SV: Stroke volume
SV/LVET: Index of contractility
SVR: Systemic vascular resistance
*W*_max_: Maximum workload achievable
